# Impact of social isolation on older outpatients during the COVID-19 pandemic

**DOI:** 10.31744/einstein_journal/2026AO1970

**Published:** 2026-09-23

**Authors:** Leonardo Trovo Zilotti, Alexandre Elias Romanos, Marcelo Kenzo Naya Takahashi, Alexandre Leopold Busse, Wilson Jacob

**Affiliations:** 1 Hospital das Clínicas Faculdade de Medicina Universidade de São Paulo São Paulo SP Brazil Hospital das Clínicas, Faculdade de Medicina, Universidade de São Paulo, São Paulo, SP, Brazil.

**Keywords:** COVID-19, Social isolation, Aged, Functional status, Pandemics, Psychological well-being, Surveys and questionnaires

## Abstract

**Objective:**

We aimed to evaluate the impact of social isolation during the coronavirus disease 2019 (COVID-19) pandemic on the health of older outpatients in São Paulo, Brazil.

**Methods:**

A retrospective cohort study was conducted using the Multidimensional Assessment of the Elderly in Primary Care (AMPI-AB) tool to assess the functionality of individuals aged 60 years and older at three time-points: pre-pandemic (January 2019 - March 2020), during the pandemic (October 2021 - August 2022), and post-pandemic (September 2022 - July 2023). Data were extracted from medical records.

**Results:**

Among the 206 patients evaluated pre-pandemic, 94 were assessed during the pandemic, with 80 included in the final analysis. The mean age was 76.7 years; participants were predominantly female (66%). The number of healthy patients decreased from 52 pre-pandemic to 37 during the pandemic (18.8% reduction, p=0.001), while post-pandemic, the number of healthy patients increased to 45 (21.6% improvement from the pandemic period, p <0.001). The average AMPI-AB score rose from 4.8 points pre-pandemic to 6.2 points during the pandemic, then slightly decreased to 6.0 points post-pandemic, indicating a modest recovery in functionality.

**Conclusion:**

Our findings highlight significant consequences of social isolation on the health of the older population, which are characterized by a decline in well-being and functionality during the COVID-19 pandemic. However, a notable recovery was observed post-pandemic, emphasizing the need for targeted interventions to support vulnerable older individuals in future public health crises.

## Highlights

■ A significant decline in functional status was observed during the COVID-19 pandemic.

■ Functional recovery was observed after the pandemic, predominantly in the pre-frail subgroup.

■ Despite the overall recovery trend, participants in the frail subgroup did not demonstrate improvement.

## In Brief

This retrospective cohort study by Zilotti et al. aimed to objectively investigate the impact of social isolation during the COVID-19 pandemic on community-dwelling older adults in the city of São Paulo, Brazil. Functional status was assessed at three distinct time-points: before, during, and after the pandemic. This longitudinal evaluation allowed the authors to analyze the functional behavior of the studied population throughout this unprecedented global health event.

## Graphical abstract

**Figure:**
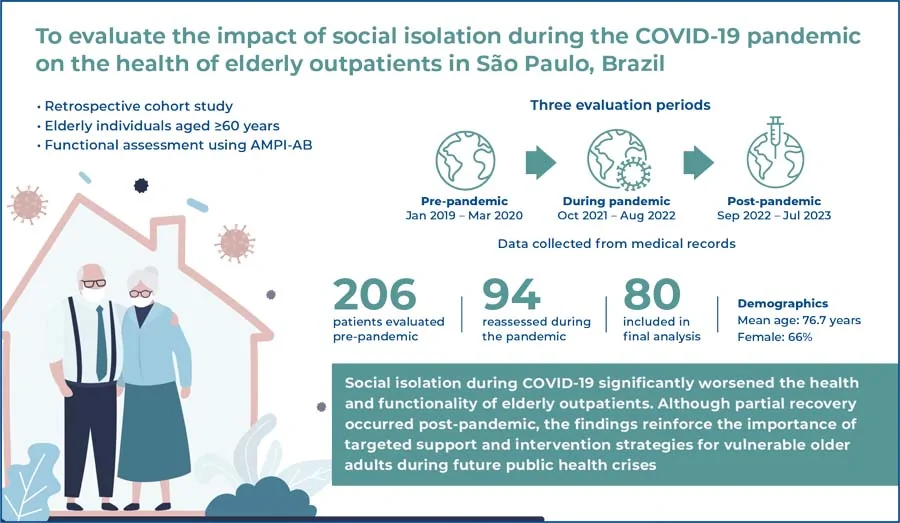

## INTRODUCTION

The coronavirus disease 2019 (COVID-19) pandemic had both direct and indirect impacts on the global population. Those who were minimally affected by the direct action of the virus were nonetheless exposed to the socio-economic changes of the period, particularly social isolation and its consequences. The older population, perceived as more vulnerable owing to initial mortality data,^(1,2)^ became the target of governmental and community policies, thus facing prolonged uncertainty, and being restricted to their homes or other familiar settings.

Much has been discussed regarding the actual necessity of establishing social isolation during this critical moment. Even today – in a post-pandemic scenario – debates continue about the true effectiveness of confinement. Regardless of its apparent success^(3)^ in partially controlling the etiological agent, it is unquestionable that it brought immediate consequences to the daily lives of all, especially older individuals. The reduction of social and family interaction, the difficulty in accessing medical/multidisciplinary care, and the restrictions on outdoor or collective physical activities are notable examples of the rapidly notable changes.

Perceptions of the deterioration of the older adults’ global health related to the isolation period are frequently reported in medical consultations. Phrases such as “my mother deteriorated significantly during the pandemic” or “I lost a lot of weight during the pandemic years” have become common during consultations. These observations lead to the interpretation of the pandemic as a frail event, yet few objective evaluations are found in the literature regarding this matter.

## OBJECTIVE

The present retrospective cohort utilized the Multidimensional Assessment of the Elderly in Primary Care (AMPI-AB) tool to evaluate the functionality of individuals aged 60 years or older during three time-points: pre-pandemic, during the pandemic, and post-pandemic.

## METHODS

This observational cohort study evaluated the impact of the pandemic and its consequent social isolation on older individuals using the AMPI-AB tool within a primary care program for such patients at a Geriatric Service of a Teaching Hospital in São Paulo.

The study was approved by the Research Ethics Committee of the *Faculdade de Saúde Pública, da Universidade de São Paulo* (CAAE: 85167124.7.0000.5421; # 7,277,617).

### Data collection and organization

Within the routine of the service where the work was performed, the AMPI-AB is applied at least annually to registered patients in the outpatient clinic. Therefore, data collection occurred retrospectively through a review of the physical medical records at the unit. In this healthcare center, the AMPI-AB tool was applied by workers previously trained for this task, such as physicians, medical interns, or members of the multidisciplinary team. Patients who had an evaluation using the AMPI-AB at three time-points–pre-pandemic, during the pandemic, and post-pandemic – were included, allowing for a detailed assessment of the functionality curve of the individuals included based on the collected data. The divisions of each of these periods were made according to the stages of the pandemic, following this chronology: the pre-pandemic period was defined as between January 2019 and March 2020, as the significant outbreak of viral cases occurred in April 2020. The pandemic period was delineated from October 2021 to August 2022, which represented the most critical moment in Brazilian territory. Finally, the post-pandemic period was defined from September 2022 to July 2023, corresponding to the easing of social isolation measures. The minimum interval between evaluations was six months: Pre-pandemic: January 2019 to March 2020 (15 months); Pandemic: October 2021 to August 2022 (11 months); Post-pandemic: September 2022 to July 2023 (11 months)

### Inclusion criteria

All individuals aged 60 years and older who underwent medical consultations during the investigated periods.

### Exclusion criteria

Individuals without AMPI-AB assessments at all three specified time-points were excluded. To ensure greater accuracy regarding the impact of the pandemic on the inclusion group, patients with evaluations prior to January 2019 were excluded to avoid long intervals relative to the onset of the pandemic in Brazil.

### AMPI-AB

AMPI-AB is a tool created in 2015 that evaluates 17 biopsychosocial parameters of older individuals. A study confirmed the validity of the method in São Paulo and demonstrated accuracy in detecting frailty, predicting mortality, loss of functionality (basic or instrumental), risk of hospitalizations, emergency visits, and falls.^(4)^ These 17 parameters include: age, self-perception of health, family support, number of chronic conditions, number of medications in use, number of hospitalizations in the last year, number of falls in the last year, visual and/or auditory impairment, physical limitation, cognitive worsening (subjective or perceived by family), mood disorder, functionality for basic and/or instrumental activities, continence, unintentional weight loss, and oral problems (swallowing). Despite appearing lengthy, the average time for administering the assessment is 5 min 44 s. The higher the score from the current assessment, the worse the functional condition is. The AMPI-AB results classify individuals into one of the following groups: healthy (<6 points), pre-frail (6–10 points), and frail (>10 points). For statistical purposes, patients will be categorized as healthy (<6 points) and unhealthy (≥6 points).

### Statistical assessment

We used descriptive statistical methods to calculate means and standard deviations for quantitative variables and absolute and relative frequencies for categorical variables. The included participants were compared across the three periods of AMPI-AB application regarding their sociodemographic characteristics and scores on the parameters evaluated by the AMPI-AB.

We used the paired Student-Welch *t*-test for continuous quantitative variables and χ^2^ and Fisher’s exact tests for categorical variables. The AMPI-AB scores were paired based on the moment of application (pre-pandemic and pandemic, pre-pandemic and post-pandemic) and compared using the paired Student-Welch *t*-test. An alpha level of 5% was considered for the tests, along with a 95% confidence interval (95%CI). The statistical software R version 4.2.2 (R Core Team, Vienna, Austria) was utilized for the analyses in RStudio IDE (Posit PBC, Boston, MA, USA).

The primary objective of the study was to assess whether the pandemic impacted the number of patients classified as healthy, as identified by AMPI-AB, while the secondary objective was to evaluate whether any of the parameters assessed by AMPI-AB had a differentiated isolated impact on the studied population (comparing intra-pandemic with post-pandemic).

## RESULTS

In a review of medical records, 206 patients were identified as having been assessed with the AMPI-AB tool during the pre-pandemic period. Of these, 109 were not re-evaluated during the pandemic period (loss of follow up or did not want to participate). Therefore, 97 patients were assessed during both the pre-pandemic and intra-pandemic periods. Among these, 3 patients passed away (from causes other than COVID), and 14 patients were lost to follow-up during the post-pandemic period. Thus, 80 patients were evaluated with the AMPI-AB before, during, and after the pandemic (n=80). Data are presented in figure 1 – não estou conseguindo colocar a forma editável do flowchart diretamente no word.

**Figure 1:**
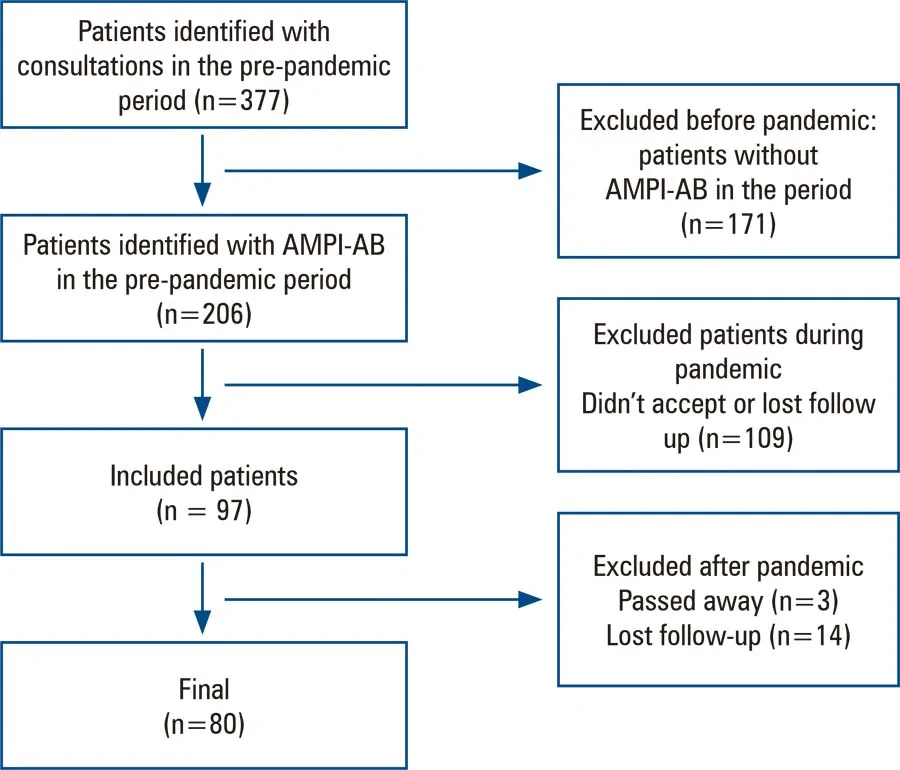
Flowchart of the final number of included individuals

The average age of the studied population was 76.7 years, with a predominance of self-reported White patients (76%) and female ones (66%). The sociodemographic characteristics are presented in table 1.

**Table 1:** Sociodemographic data of participants (n=80)

Age (years), mean (standard deviation)	76.7 (7.2)
Women, n (%)	53 (66.2)
Self-declared race, n (%)	
White	61 (76.2)
Yellow	10 (12.5)
Brown	6 (7.5)
Black	2 (2.5)
Not declared	1 (1.2)

The participant’s clinical variables were not presented in table 1 because the “number of chronic conditions” constitutes one of the domains assessed by the study’s research instrument (AMPI-AB). At baseline, 35 participants (43%) had 1–2 chronic conditions and 32 (40%) had three or more chronic conditions. Regarding medication use, 45 participants (56.3%) were taking 1–4 medications daily, whereas 35 participants (43.8%) were taking five or more medications per day.

In the evaluation of the categorical variable (Table 2), 52 patients were considered healthy before the pandemic and 28 unhealthy (27 pre-frail and 1 frail). During the pandemic, the group of healthy patients decreased by 18.8% (from 52 to 37 patients, p=0.001) with partial recovery in the post-pandemic scenario (from 37 to 45 members, p<0.001). The continuous variable (mean score on the AMPI-AB tool) was 4.8 points before the pandemic, increasing to 6.2 points during the pandemic, with a slight variation to 6.0 (-0.2) points in the post-pandemic scenario.

**Table 2:** Classification and Scoring in AMPI-AB (n=80)

Variables	Pre-pandemic	During the pandemic	p value	After the pandemic	p value
Classification, n (%)			0.001		<0.001
Healthy	52 (65)	37 (46.2)		45 (56.3)	
Unhealthy	28 (35)	42 (53.8)		35 (43.7)	
Pre-frail	27 (33.4)	36 (45)		25 (31.3)	
Frail	1 (1.3)	7 (8.8)		10 (12.5)	
Mean score (SD)	4.8 (2.3)	6.2 (3.1)	<0.001	6.0 (3.5)	0.312
			difference: 1.46 95%CI= (0.84, 2.08)		difference: 0.24 95%CI= (-0.23, 0.70)

Although the post-pandemic scenario showed partial recovery in the health status of the studied population (Figure 2), the continuous variable remained stable when comparing the intra-pandemic with the post-pandemic period. This difference may be explained by the difficulty in recovery among unhealthy patients in the “frail” subgroup. Of the 7 patients who were classified as frail during the pandemic, none returned to the healthy group. The pre-frail group, on the other hand, saw a reduction of 11 representatives.

The three deaths that occurred during the pandemic were not due to factors directly related to the coronavirus and were instead attributed to colorectal cancer and urinary sepsis. In the third case the family was unaware of the cause of death but denied COVID infection.

In the evaluation of the isolated domains of the AMPI-AB tool, only 2 of the 17 parameters showed statistical relevance: the number of hospitalizations and dependence on basic activities of daily living (ADL). Hospitalizations increased after the easing of social isolation, as 7 patients were hospitalized during the pandemic and 14 after the pandemic (p=0.015). The number of patients dependent on ADL decreased from 5 (during the pandemic) to 3 in the post-pandemic scenario (p=0.009). The table with the isolated evaluation of each parameter is available as supplementary material.

## DISCUSSION

In the studied older population, the social isolation caused by the pandemic had significant consequences. Since the usual frail rate of this population was not previously defined, it is not possible to compare it with the reduction of 15 patients (from 52 to 37) found in the healthy group, which we consider to be an alarming variation. The impact of the pandemic becomes even more evident considering the partial recovery of the healthy group after the easing of social isolation (from 37 to 45 patients).

The correlation between frailty and social isolation is well-established in the literature.^(5)^ Among the theories attempting to explain this association, several studies demonstrate that social restriction is linked to unhealthy behaviors, such as poor diet, tobacco or alcohol abuse, and physical inactivity.^(6)^ The increase in inflammatory markers and interleukins in patients exposed to this scenario has also been studied. C-reactive protein and interleukin-6, which are associated with poorer physical performance and cardiovascular disease, are found at higher levels in individuals experiencing isolation.^(7)^

More recently, in 2024, social exclusion has also been recognized as a modifiable risk factor for dementia.^(8)^ All these recent findings regarding the topic have led the World Health Organization to view social isolation and loneliness as a threat to global health, making it a priority issue for the “Decade of Healthy Aging.”

Despite the aforementioned points, we can assert that the social isolation during the pandemic had very specific characteristics. First, it was not voluntary social isolation but rather a public health measure. Second, it occurred during an unprecedented time filled with fear and uncertainty. Third, at various moments, health, leisure, and commercial services faced activity restrictions. Therefore, although it represents a tragic chapter in human history, it also provided an opportunity to study a unique epidemiological moment – which, in our view, adds greater relevance to this study.

We cannot predict when we will face another pandemic situation, but it is essential to have a clear understanding of the scientific evidence that can guide our decision-making in future responses.

Despite the partial recovery of the studied population, the subgroup of frail individuals in the post-pandemic period exhibited significantly concerning behavior: of the seven patients who became frail during the period of social isolation, all remained frail in the post-pandemic period. Conversely, the 36 pre-frail patients during the pandemic decreased to 25 in the post-pandemic phase. This finding aligns with the previously established knowledge regarding the difficulty of recovering a patient from frailty.^(9,10)^ Even when this group is exposed to rehabilitation therapies, they demonstrate lower resilience compared to pre-frail and healthy patients. Pre-frailty should indeed be faced as a therapeutic window of opportunity.^(11)^

The consequence of this condition raises important questions: “Are we adequately treating individuals with frailty?,” “Should we intensify our nutritional and physical approaches?,” or “Is the progression from frailty to death an inexorable trajectory in aging complicated by multimorbidity?” Studies are already emerging that articulate the question: “Is it time to initiate public campaigns on pre-frailty and frailty?”^(12)^

When evaluating the isolated parameters of the AMPI-AB, the two that showed statistical significance were the increase in the number of hospitalizations and functionality for ADL, which appears paradoxical. How can a population that improves its functionality require a greater number of hospital admissions? In our opinion, during the pandemic, healthcare services were almost restricted to the treatment of patients infected with COVID-19, resulting in a decreased capacity to address other health issues, especially for those requiring elective hospitalizations. Eventually, following the easing of social isolation, access to hospitals was facilitated, leading to the attention of a previously unmet demand.

## CONCLUSION

This study has some limitations, among which we highlight the following: as this was an observational study, it could only generate hypotheses; being conducted at a single primary healthcare unit limits the generalizability of these results to the Brazilian Public Health System as a whole; and the significant exclusion of patients from our sample may have introduced a selection bias. Nonetheless, we believe that the results presented and discussed here, from this unique moment in global health, demonstrate a relationship between the social isolation generated by the pandemic and the variation in functionality of older patients before, during, and after the pandemic.
